# Predicting Disease-Specific Survival for Patients With Primary Cholangiocarcinoma Undergoing Curative Resection by Using a Decision Tree Model

**DOI:** 10.3389/fonc.2022.824541

**Published:** 2022-04-21

**Authors:** Bing Quan, Miao Li, Shenxin Lu, Jinghuan Li, Wenfeng Liu, Feng Zhang, Rongxin Chen, Zhenggang Ren, Xin Yin

**Affiliations:** ^1^ Liver Cancer Institute, Zhongshan Hospital, Fudan University, Shanghai, China; ^2^ National Clinical Research Center for Interventional Medicine, Zhongshan Hospital, Fudan University, Shanghai, China

**Keywords:** cholangiocarcinoma, resection, disease-specific survival, prediction, decision tree

## Abstract

**Background:**

The aim of this study was to derive and validate a decision tree model to predict disease-specific survival after curative resection for primary cholangiocarcinoma (CCA).

**Method:**

Twenty-one clinical characteristics were collected from 482 patients after curative resection for primary CCA. A total of 289 patients were randomly allocated into a training cohort and 193 were randomly allocated into a validation cohort. We built three decision tree models based on 5, 12, and 21 variables, respectively. Area under curve (AUC), sensitivity, and specificity were used for comparison of the 0.5-, 1-, and 3-year decision tree models and regression models. AUC and decision curve analysis (DCA) were used to determine the predictive performances of the 0.5-, 1-, and 3-year decision tree models and AJCC TNM stage models.

**Results:**

According to the fitting degree and the computational cost, the decision tree model derived from 12 variables displayed superior predictive efficacy to the other two models, with an accuracy of 0.938 in the training cohort and 0.751 in the validation cohort. Maximum tumor size, resection margin, lymph node status, histological differentiation, TB level, ALBI, AKP, AAPR, ALT, γ-GT, CA19-9, and Child-Pugh grade were involved in the model. The performances of 0.5-, 1-, and 3-year decision tree models were better than those of conventional models and AJCC TNM stage models.

**Conclusion:**

We developed a decision tree model to predict outcomes for CCA undergoing curative resection. The present decision tree model outperformed other clinical models, facilitating individual decision-making of adjuvant therapy after curative resection.

## Introduction

Cholangiocarcinoma (CCA) is a rare malignancy that originates from anywhere along the bile ducts and/or within the hepatic parenchyma (1). During these years’ examinations, incidence rates of CCA increased in most countries investigated worldwide (2). CCA is classified as intrahepatic CCA (iCCA), perihilar CCA (pCCA), and distal CCA (dCCA) according to the primary anatomic subtypes (3, 4). Surgical treatments, including partial hepatectomy (PHx), hilar resection with extended hepatectomy, local bile duct resection, or pancreatoduodenectomy (PD), remain the gold standard for CCA (5). Despite advances in comprehensive preoperative patient selection, surgical techniques, as well as perioperative care, patients with CCA have poor prognosis. More than half of patients present with tumor recurrence within 2 years after curative resection, and a considerable number of patients will develop tumor recurrence as early as 6 months (6, 7). Overall, patients who undergo curative-intent resection have a median overall survival (OS) range from 15 to 30 months (5, 8, 9).

In most countries examined, the incidence of iCCA was higher than pCCA. It is posited that the true incidence of iCCA was significantly overestimated owing to the extensive misclassification of pCCA as iCCA (10–12). To date, several clinical models have been developed to predict disease-specific survival (DSS) in CCA (5, 8, 13–17). However, these currently available models targeted only one subtype of CCA; the inevitable misclassification of pCCA and iCCA may have effect on the accuracy of these models.

The decision tree model is a predictive tool that uses both categorical and numerical data aiming at assigning samples to specific classes. Unlike models such as artificial neural networks (ANNs), the thresholds and class predictions calculated by the decision tree model often have practical interpretation that can be used to provide intuitive decision-making for clinicians (18). Meanwhile, the decision tree model is particularly well suited for small sample sizes of the database. Regression model is widely used and easy to understand, but too simple to capture complex relationships (19). However, few studies have compared the performance of the decision tree model with regression model in CCA.

Given that DSS can specifically reflect tumor-associated prognosis, the aim of our study is to predict DSS for patients with primary CCA (including iCCA, pCCA, and dCCA) after curative resection by using the decision tree model, generated by machine learning algorithm. We hope our model can help to predict individual prognosis and shed light on clinical decision-making for patients with primary CCA.

## Patients and Methods

### Study Population

From January 1995 to December 2014, patients who underwent curative resection for primary CCA in the Liver Cancer Institute, Zhongshan Hospital were retrospectively screened. The inclusion criteria were as follows: (1) pathologically confirmed as primary CCA by curative resection; (2) Child-Pugh grade A or B; and (3) with detailed preoperative clinical characteristics and prognostic data. The exclusion criteria were as follows: (1) with other malignant diseases in addition to CCA; (2) with positive surgical R2 margins confirmed by pathology; (3) re-resection for tumor recurrence; and (4) accidental deaths due to other diseases. Adjuvant chemotherapy or other systematic therapies were used following surgical resection when people still had positive regional node involvement or microscopic margins according to the NCCN guidelines. This study was performed in accordance with the Medical Ethics Committee of Zhongshan Hospital affiliated to Fudan University (Approval NO: B2021-775R) and the Helsinki Declaration.

### Data Collection

The clinical characteristics were collected for each patient, including age, sex, cirrhosis, comorbid illness, TB (total bilirubin) level, AKP (alkaline phosphatase), ALT (alanine aminotransferase), AST (alkaline phosphatase), albumin level, γ-GT (γ-glutamyl transpeptidase), CA19-9 (carbohydrate antigen 19-9), Child-Pugh grade, maximum tumor size, tumor number, tumor location, endovascular embolization, BDT (bile duct thrombi), resection margin, resection procedure, lymph node metastasis, histological differentiation, and DSS. Comorbid illnesses included diabetes mellitus, hypoglycemia, and erythrocytosis. AKP negative was defined as ≤125 in male patients and ≤135 in female patients, while AKP positive was defined as >125 in male patients and >135 in female patients. Tumor location included iCCA, pCCA, and dCCA. Major resection was defined as resection of 3 or more liver segments (20), while minor resection was defined as 2 or less liver segments and bile duct resection alone. We divided people whose lymph node metastasis were Nx into low risk and high risk according to clinical risk score (CRS) (21) [CRS = 1.76 − 0.022 × Age + 0.132 × Number of lesions + 0.645 * CA19-9 (>200: 1, ≤200: 0) + 0.333 ALBI grade (grade 2/3: 1, grade 1: 0)]. DSS was defined as the time interval between the date of resection for primary CCA and the date of death caused by related disease or last follow-up.

### Follow-Up

All patients were followed up every 2–3 months in the first year after surgical resection, and then every 6 months in the following years until death or dropout. Physical examinations, routine blood tests, liver function tests, and tumor marker tests were performed routinely. Chest radiography, computed tomography (CT), magnetic resonance imaging (MRI), bone scan, and brain CT scan were performed when recurrence or distant metastasis was suspected.

### Statistical Analysis

Continuous variables were expressed as median with interquartile range (IQR) and compared using the Student’s *t*-test or the Mann–Whitney *U* test. Categorical variables were expressed as counts with percentages and compared using the *χ* ^2^ test or the Fisher’s exact test. The independent factors for predicting DSS after curative resection for primary CCA were identified using univariable and multivariable Cox regression analyses, and those variables found significant at *p* < 0.05 in univariable analyses were entered into multivariable analyses. According to the four independent factors, survival differences among groups were examined by Kaplan–Meier analysis and log-rank test. Notably, we divided maximum tumor size into four groups (<2.0 cm, 2.0–5.0 cm, 5.0–10.0 cm, and ≥10.0 cm) in this step. The statistical analysis was performed using SPSS 26.0 and R 3.5.1.

### Decision Tree Model

On a computer with an Intel (R) Core (TM) i5-8250U CPU running Python 3.7.10, we deployed a decision tree model to aggregate baseline risk factors and to predict the probability of survival using the package “tree” and “metrics” of Scikit Learn. First, a total of 482 patients of primary CCA were enrolled and we manipulated data using the package “pandas” and “numpy”. Then, we randomly divided 482 patients into the training cohort (*n* = 289) and the validation cohort (*n* = 193) using the package “model_selection” of Scikit Learn. In our study, variables identified as important factors in the univariable and multivariable analysis and all 21 clinical variables were entered into decision tree models, respectively. We searched the best decision tree model from three models according to the accuracy and the computational cost. The accuracy was calculated by the formula: (TP+TN)/(P+N). The computational cost was defined as the time from model establishment to model calculation. Gini index was defined as the criterion of the classification for the decision tree model. The predictive performance of the decision tree models and regression models were compared in both training cohort and validation cohort by calculating receiver operating characteristic (ROC), sensitivity, and specificity. In addition, we also compared the predictive performance of decision tree models with that of AJCC (American Joint Committee on Cancer) TNM stage models by using ROC analysis as well as decision curve analysis (DCA), since DCA was more informative than area under the curve (AUC) in estimating the clinical value of a model (22, 23). Notably, we merged the two cohorts together in this step. The statistical analysis was performed using SPSS 26.0 and python 3.9.2.

## Results

### Patients and Survival Outcomes

The training cohort consisted of 289 consecutive eligible patients who had received resection for primary CCA. The validation cohort consisted of 193 consecutive eligible patients. The clinical characteristics of the patients were presented in **Table 1**. There were no significant differences in the two cohorts. In the training cohort, most patients had iCCA (252 [87.2%]), and a subset of patients had pCCA (29 [10.0%]) and dCCA (8 [2.8%]). In the validation cohort, patients had iCCA (171 [88.6%]), pCCA (14 [7.3%]), and dCCA (8 [4.1%]). The median (IQR) DSS was 16.0 (9.0–27.5) months and the 0.5-, 1- and 3-year DSS rates were 81.3%, 60.2%, and 14.9% in the training cohort. The median (IQR) DSS was 15.0 (8.0–31.5) months and the 0.5-, 1-, and 3-year DSS rates were 80.3%, 54.9%, and 20.2% in the validation cohort.

**Table 1:** Clinical characteristics of the study population.

Variables	Number (proportion, %) or median (IQR)		*p*
	The training group (*n* = 289)	The testing group (*n* = 193)	
Age, ≥60/<60 (years)	121/168 (41.9/58.1)	69/124 (35.8/64.2)	0.463
Sex, Male/Female	173/116 (59.9/40.1)	123/70 (63.7/36.3)	0.026
Cirrhosis, Yes/No	10/279 (3.5/96.5)	14/179 (7.3/92.7)	0.782
Comorbid illness, Yes/No	16/273 (5.5/94.5)	12/181 (6.2/93.8)	0.205
TB level (μmol/L)	12.7 (8.9–18.2)	11.6 (9.3–15.6)	0.420
AKP (U/L)	100.5 (74.3–167.3)	94.0 (71.0–136.0)	0.405
ALT (U/L)	29.0 (18.0–46.0)	26.0 (17.0–42.0)	0.492
AST (U/L)	30.5 (22.0–44.0)	28.5 (22.0–47.3)	0.565
Albumin level (g/L)	43.0 (39.0–46.0)	44.0 (40.3–47.0)	0.305
γ-GT (U/L)	71.5 (37.0–136.8)	68.5 (37.0–142.0)	0.363
CA19-9 (kU/L)	33.4 (13.5–157.4)	39.8 (11.8–213.6)	0.537
Child-Pugh grade, A/B	264/25 (91.3/8.7)	178/15 (92.2/7.8)	0.239
Maximum tumor size (cm)	6.0 (3.0–11.0)	6.0 (3.0–8.0)	0.473
Tumor number, Multiple/Solitary	75/214 (26.0/74.0)	63/130 (32.6/67.4)	0.061
Endovascular embolization, Yes/No	108/181 (37.4/62.6)	84/109 (43.5/56.5)	0.939
BDT, Yes/No	6/283 (2.0/98.0)	4/189 (2.1/97.9)	0.174
Resection margin, R1/R0	26/263 (9.0/91.0)	12/181 (6.2/93.8)	0.144
Resection procedure, Major/Minor	132/157 (45.7/54.3)	80/113 (41.5/58.5)	0.621
Lymph node metastasis, Nx (low risk)/Nx (high risk)/N0/N1	30/44/178/37 (10.4/15.2/61.6/12.8)	25/35/108/25 (13.0/18.1/56.0/32.6)	0.245
Histological differentiation, Well or Moderate/Poor	180/101 (65.1/34.9)	130/63 (67.4/32.6)	0.299
Overall survival (months)	16.0 (9.0–27.5)	15.0 (8.0–31.5)	0.948

TB, total bilirubin; AKP, alkaline phosphatase; ALT, alanine aminotransferase; AST, alkaline phosphatase; γ-GT, γ-glutamyl transpeptidase; CA19-9, carbohydrate antigen 19-9; BDT, bile duct thrombi.

### Univariable and Multivariable Analysis

**Figure 1:**
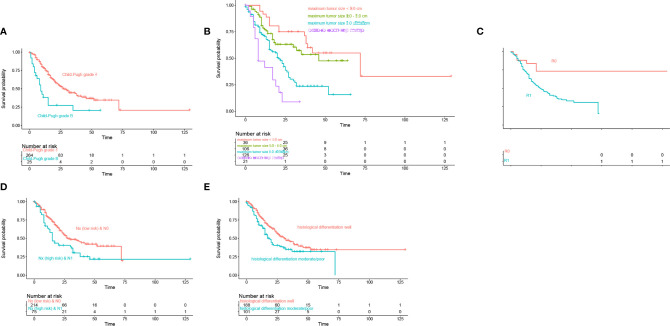
Kaplan-Meier curves estimate of overall survival according to **(A)** Child-Pugh grade, **(B)** Maximum tumor size, **(C)** Resection margin, **(D)** Lymph node status, **(E)** Histological differentiation.

In the training cohort of 289 patients, TB level (>17.1 vs. ≤17.1), ALBI (1 vs. 2 vs. 3), AKP (Positive vs. Negative), AAPR (≤0.4 vs. > 0.4), albumin level (<35 vs. ≥35 g/L), γ-GT (>50 vs. ≤50 U/L), CA19-9 (>37 vs. ≤37 kU/L), Child-Pugh grade (B vs. A), maximum tumor size (Continuous variable, cm), lymph node status [Nx (low risk) vs. Nx (high risk) vs. N0 vs. N1], and histological differentiation (Well vs. Moderate/Poor) were related to DSS in the univariable Cox regression analysis (**Table 2**). The multivariable Cox regression analysis confirmed AAPR (≤0.4 vs. >0.4), maximum tumor size (cm), lymph node status [Nx (low risk) vs. Nx (high risk) vs. N0 vs. N1], and histological differentiation (Well vs. Moderate/Poor) as independent predictors (**Table 2**). Meanwhile, Kaplan–Meier curves suggested that Child-Pugh grade A, small tumor size, R0 resection, lymph node status [Nx (low risk) and N0], and well histological differentiation correlated with better survival outcomes in **Figures 1A–E**.

**Table 2:** Univariable and multivariable Cox regression analysis of predicting outcomes in patients after resection for cholangiocarcinoma in the training group.

Variables	OR Comparison	UV OR (95% CI)	UV *p*	MV OR (95% CI)	MV p*
Age	≥60 vs. <60 years	1.162 (0.833–1.620)	0.377		
Sex	Male vs. Female	0.893 (0.637–1.252)	0.511		
Cirrhosis	Yes vs. No	0.718 (0.265–1.942)	0.514		
Comorbid illness	Yes vs. No	0.500 (0.204–1.222)	0.129		
TB level	>17.1 vs. ≤17.1	1.467 (1.017–2.116)	**0.041**	1.306 (0.728–2.342)	0.370
ALBI	1	1.000 (reference)		1.000 (reference)	
	vs. 2	1.545 91.057–2.258)	**0.025**	0.860 (0.515–1.435)	0.564
	vs. 3	4.477 (1.817–11.032)	**0.001**	1.525 (0.478–4.865)	0.475
†AKP	Positive vs. Negative	1.897 (1.341–2.683)	**<0.001**	1.205 (0.669–2.171)	0.535
AAPR	≤0.4 vs. >0.4	1.890 (1.354–2.637)	**<0.001**	1.285 (0.789–2.092)	0.314
ALT	>50 vs. ≤50 U/L	1.548 (1.046–2.291)	**0.029**	1.484 (0.874–2.520)	0.144
AST	>40 vs. ≤40 U/L	1.390 (0.951–2.032)	0.089		
Albumin level	<35 vs. ≥35 g/L	1.376 (0.976–1.940)	0.069		
γ-GT	>50 vs. ≤50 U/L	1.781 (1.253–2.531)	**0.001**	1.103 (0.739–1.647)	0.631
CA19-9	>37 vs. ≤37 kU/L	2.130 (1.510–3.005)	**<0.001**	1.476 (0.999–2.181)	0.051
Child-Pugh grade	B vs. A	2.473 (1.503–4.069)	**<0.001**	2.061 (1.001–4.242)	**0.050**
Maximum tumor size	Continuous variable, cm	1.203 (1.142–1.267)	**<0.001**	1.297 (1.217–1.382)	**< 0.001**
Tumor number	Continuous variable	0.948 (0.850–1.056)	0.333		
Endovascular embolization	Yes vs. No	0.972 (0.693–1.362)	0.867		
Resection margin	R1 vs. R0	2.577 (1.136–5.843)	**0.023**	2.785 (1.168–6.641)	**0.021**
Resection procedure	Major vs. Minor	0.942 (0.674–1.316)	0.727		
Lymph node status	Nx (low risk) and N0 vs. Nx (high risk) and N1	1.696 (1.199–2.397)	**0.003**	2.157 (1.468–3.170)	**< 0.001**
Histological differentiation	Well vs. Moderate/Poor	1.524 (1.089–2.133)	**0.014**	2.007 (1.378–2.925)	**< 0.001**

*Those variables found significant at p < 0.05 in univariable analyses were entered into multivariable analyses.

†AKP negative is defined as ≤125 in male and ≤135 in female. AKP positive is defined as >125 in male and >135 in female.

OR, odds ratio; UV, univariable; MV, multivariable; CI, confidence interval; TB, total bilirubin; ALBI, albumin-bilirubin; AKP, alkaline phosphatase; AAPR, albumin-to-alkaline phosphatase ratio; ALT, alanine aminotransferase; AST, alkaline phosphatase; γ-GT, γ-glutamyl transpeptidase; CA19-9, carbohydrate antigen 19-9; BDT, bile duct thrombi. P values which < 0.05 in univariable analyses and multivariable analyses were indicated in bold text.

### Establishment of the Decision Tree Model

**Figure 2:**
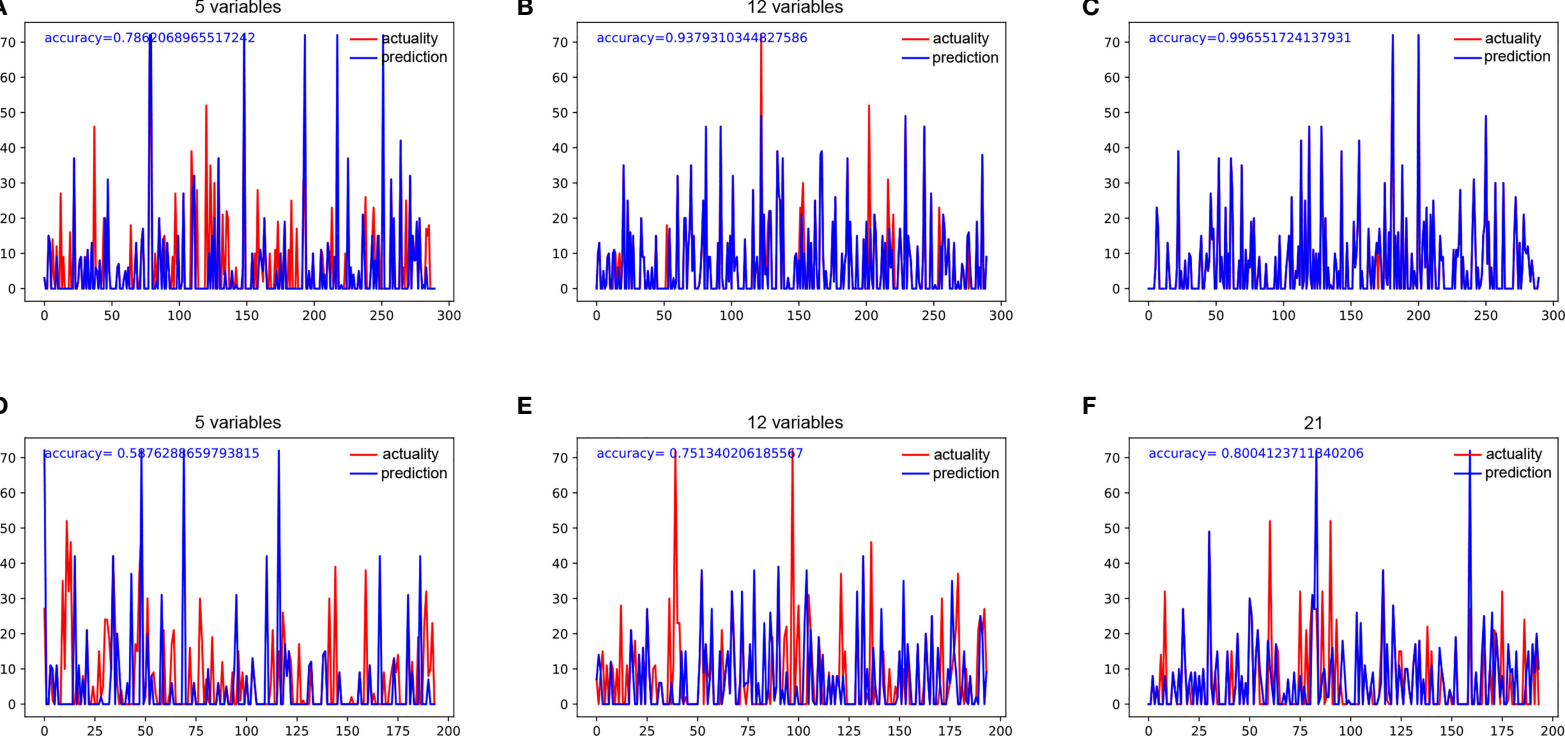
**(A)** The accuracy of decision tree analysis based on 5 variables in the training group. **(B)** The accuracy of decision tree analysis based on 12 variables in the training group. **(C)** The accuracy of decision tree analysis based on 21 variables in the training group. **(D)** The accuracy of decision tree analysis based on 5 variables in the testing group. **(E)** The accuracy of decision tree analysis based on 12 variables in the testing group. **(F)** The accuracy of decision tree analysis based on 21 variables in the testing group.

**Figure 3:**
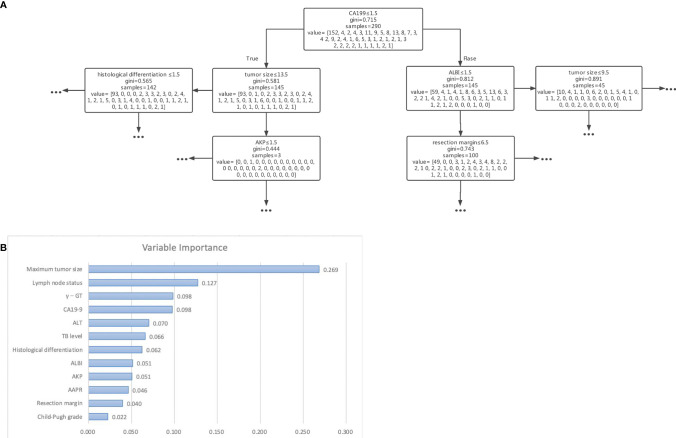
**(A)** Schematic representation of the decision tree analysis based on 12 variables used to predict outcomes in patients after resection for cholangiocarcinoma. **(B)** The importance of each variable in the decision tree analysis based on 12 variables.

We used 5 independent predictors, 12 predictors in the univariable Cox regression analysis, and all 21 clinical characteristics to build three decision tree models, respectively. The accuracy of decision tree models based on 5 variables, 12 variables, and 21 variables in the training cohort were 0.786, 0.938, and 0.997 (**Figures 2A–C**), respectively. The accuracy of decision tree models based on 4 variables, 12 variables, and 21 variables in the training cohort were 0.588, 0.751, and 0.800 (**Figures 2D–F**), respectively. The results indicated that the decision tree model based on 12 variables was relatively appropriate in terms of the accuracy as well as the computational cost. The schematic representation of the decision tree model is illustrated in **Figure 3A**. The points of each risk factor in the decision tree model for the maximum tumor size, lymph node status, γ-GT, CA19-9, ALT, TB level, histological differentiation, ALBI, AKP, AAPR, resection margin, and Child-Pugh grade were 0.269, 0.127, 0.098, 0.098, 0.070, 0.066, 0.062, 0.051 0.051, 0.046, 0.040, and 0.022, respectively (**Figure 3B**).

### Predictive Performance of the Decision Tree Model and Other Models

**Figure 4:**
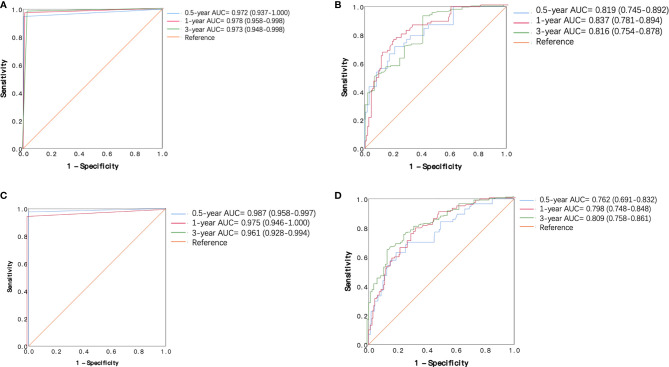
Receiver operating characteristic (ROC) curves of the decision tree model and regression model in the training cohort and validation cohort. **(A)** ROC curves of decision tree model in the training cohort. **(B)** ROC curves of regression models in the training cohort. **(C)** ROC curves of decision tree model in the validation cohort. **(D)** ROC curves of regression model in the validation cohort.

**Figure 5:**
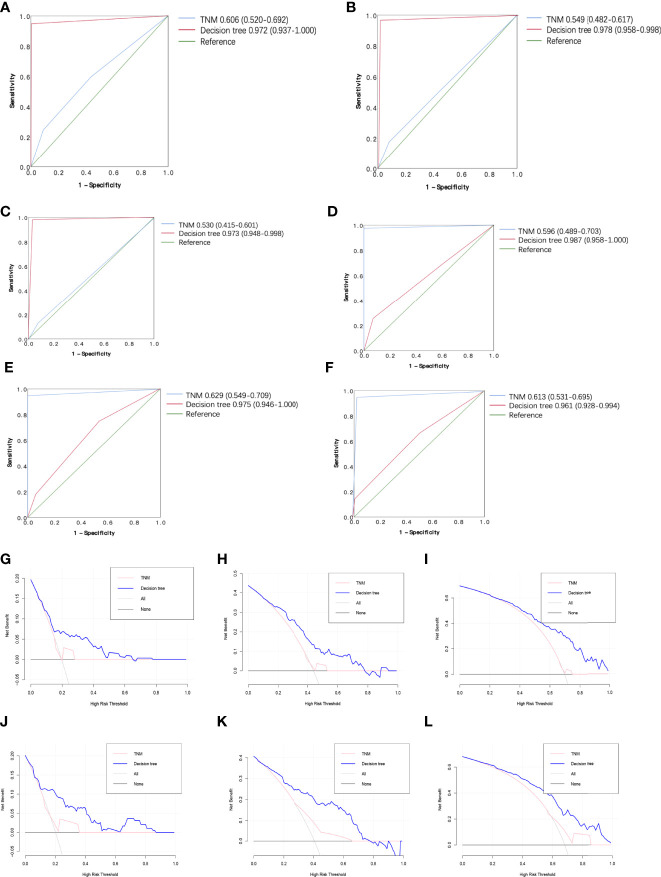
Receiver operating characteristic (ROC) curve and the decision curve analysis (DCA) of the decision tree model and AJCC TNM stage model. **(A)** 0.5-year R OC curves of the decision tree model and AJCC TNM stage model in the training cohort. **(B)** 1-year ROC curves of the decision tree mod el and AJCC TNM stage model in the training cohort. **(C)** 3-year ROC curves of the decision tree model and AJCC TNM stage model in the training cohort. **(D)** 0.5-year ROC curves of the decision tree model and AJCC TNM stage mod el in the validation cohort. **(E)** 1-year ROC curves of the decision tree model and AJ CC TNM stage model in the validation cohort. **(F)** 3-year R OC curves of the decision tree model and AJCC TNM stage model in the validation cohort. **(G)** 0.5-year DCA of the decision tree model and AJCC TNM stage model i n the training cohort. **(H)** 1-year DCA of the decision tree model and AJCC TNM stage model in the training cohort. **(I)** 3-year DCA of the decision tree model and AJCC TNM stage model in the training cohort. **(J)** 0.5-year DCA of the decision tree model and A J CC TNM stage model in the validation cohort. **(K)** 1-year DCA of the decision tree model and AJCC TNM stage model in the validation cohort. **(L)** 3-year DCA of the decision tree model and AJCC TNM stage mod el in the validation cohort.

The 0.5-, 1-, and 3-year ROC curves of the decision tree models and regression models in the training cohort and validation cohort are plotted in **Figures 4A–D**. In the training cohort, the predictive performances of the decision tree models were superior to regression models, with 0.5-, 1-, and 3-year AUC of 0.972 (0.937–1.000), 0.978 (0.958–0.998), and 0.973 (0.948–0.998), versus 0.5-, 1-, and 3-year AUC of 0.819 (0.745–0.892), 0.837 (0.781–0.894), and 0.816 (0.754–0.878), respectively. Similarly, in the validation cohort, the performance of the decision tree model was more favorable than regression models with 0.5-, 1-, and 3-year AUC of 0.987 (0.958–0.997), 0.975 (0.946–1.000), and 0.961 (0.928–0.994), versus 0.5-, 1-, and 3-year AUC of 0.762 (0.691–0.832), 0.798 (0.748–0.848), and 0.809 (0.758–0.861), respectively. **Table 3** shows that our decision tree model displayed higher sensitivity and specificity than that of the regression model in both the training cohort and the validation cohort. In addition, the predictive ability of our decision tree models was superior to the AJCC TNM stage models by using 0.5-, 1-, and 3-year AUC analysis and DCA analysis (**Figures 5A–L**).

**Table 3:** Performance indexes of decision tree analyses and regression models in the training group and testing group.

			AUC (95% CI)	Sensitivity (95% CI), %	Specificity (95% CI), %
The training cohort	0.5 years	Decision tree analysis	0.972 (0.937–1.000)	94.7 (84.5–98.6)	99.6 (97.3–100.0)
		Regression model	0.819 (0.745–0.892)	28.2 (15.6–45.1)	98.1 (94.0–99.5)
	1 year	Decision tree analysis	0.978 (0.958–0.998)	96.9 (91.6–99.0)	98.8 (95.2–99.8)
		Regression model	0.837 (0.781–0.894)	60.8 (49.1–71.4)	86.1 (78.1–91.6)
	3 years	Decision tree analysis	0.973 (0.948–0.998)	98.0 (94.7–99.4)	96.6 (89.7–99.1)
		Regression model	0.816 (0.754–0.878)	87.1 (80.0–92.1)	41.9 (29.7–55.1)
The validation cohort	0.5 years	Decision tree analysis	0.987 (0.958–0.997)	80.6 (61.9–91.9)	1.000 (0.970–1.000)
		Regression model	0.762 (0.691–0.832)	19.3 (10.5–32.3)	98.3 (95.4–99.4)
	1 year	Decision tree analysis	0.975 (0.946–1.000)	94.9 (86.9–98.4)	1.000 (0.96.0–1.000)
		Regression model	0.798 (0.748–0.848)	59.8 (50.8–68.3)	79.8 (72.6–85.5)
	3 years	Decision tree analysis	0.961 (0.928–0.994)	95.5 (89.9–98.1)	96.8 (87.8–99.4)
		Regression model	0.809 (0.758–0.861)	87.1 (81.5–91.3)	47.7 (37.1–58.6)

RF, random forest; AUC, area under the curve; CI, confidence interval.

## Discussion

In the present study, a decision tree model was developed and validated to predict DSS for patients with primary CCA undergoing curative resection. The variables included maximum tumor size, resection margin, lymph node status, histological differentiation, TB level, ALBI, AKP, AAPR, ALT, γ-GT, CA19-9, and Child-Pugh grade. Most of these risk factors were reported in previous studies and some even were involved in the similarly available CCA predictive models (8, 24–26). We found that the decision tree model outperformed the traditional regression model and the AJCC TNM stage model, contributing to prognostic prediction in patients with CCA.

The decision tree model has a well-documented history in the medical and healthcare fields for more than 30 years (27, 28). Decision trees are well suited to draw medical conclusions to support clinical decisions by using a mixture of categorical and continuous variables. Many experts were surprised at its effectiveness and accuracy of classification, and its power to provide suggestion of a decision and make an intuitive and straightforward explanation of how the decision was made at the same time. Using preoperative variables, several currently available models have been developed to access the prognosis after curative resection for CCA (5, 8, 13–17). However, most of them used nomogram and score systems. To our knowledge, this is the first study to use a decision tree model in predicting prognosis for patients with CCA undergoing curative resection.

In this study, we built three decision tree models with different numbers of variables, which were based on 5 independent predictive factors for DSS in the multivariable analysis, 12 predictive factors for DSS in the univariable analysis, and all 21 recorded factors. By applying these models, we considered all available factors that have generally been ignored in previous studies and evaluated possible combinations of various variables to identify a model with better predictive ability. Although the decision tree model with 21 variables had the best performance, it was not the appropriate choice in practice. We found that with the number of variables increasing from 5 to 12, the accuracy of the decision tree model increased by 0.152 in the training cohort and 0.163 in the validation cohort, while with the number of variables increasing from 12 to 21, the accuracy simply increased by 0.059 in the training cohort and 0.049 in the validation cohort. Obviously, the added 9 features have little contribution to the predictive ability of the model. They not only had almost no effects on the patients’ DSS, but also increased the computational cost by 50%, which greatly increased the risk of overfitting. Therefore, we recommended the decision tree model with 12 variables for clinical usage.

The AJCC TNM stage model and traditional Cox regression model are popular in cancer prognosis research. However, these models miss specific variables related to a certain disease and lack flexibility when physicians tailor prognostication for individuals. Previous studies have indicated that the accuracy of the AJCC TNM stage model for CCA is questionable, especially for pCCA and iCCA (29–31). Since traditional predictive models have difficulties dealing with non-linear relationship and overmuch variables in prognostic studies, new powerful models are warranted to fill in this gap. Our newly established model overcame the above-mentioned problems and displayed better predictive capabilities in terms of AUC analysis as well as DCA analysis.

CCA has three subtypes, namely, iCCA, pCCA, and dCCA (3, 4). Currently, to our knowledge, all available existing models merely targeted one subtype of CCA. Previous versions of the International Classification of Diseases of Oncology (ICD-O) did not include a separate code for pCCA and misclassified pCCA as iCCA extensively. The changes in ICD-O coding over time have interpreted the rising of iCCA and the declining of pCCA (10, 32, 33). It was reported that there was a trend for survival benefits in the iCCA and pCCA, compared to the dCCA (5). Therefore, we built a model that does not distinguish the location of the tumor in order to avoid the possible impact due to the misclassification of pCCA and iCCA.

There are several limitations in our study. First, despite the large sample size, this is a single-center study with a retrospective nature; multicentric or well-designed prospective studies are necessary to confirm the validation of a decision tree model. Secondly, we established our model on Chinese patients, most of whom were complicated with HBV infection, whereas different etiological backgrounds such as hepatitis C virus (HCV) infection or primary sclerosing cholangitis (PSC) were not specifically evaluated. Finally, it remains to be determined whether this model can be applied to patients who received liver transplantation for CCA (34).

In conclusion, the newly developed decision tree model can accurately predict prognosis for patients with CCA undergoing curative liver resection. Our model outperformed the AJCC TNM staging model and traditional regression models, contributing to prognosis prediction and clinical decision-making for CCA. Further validation studies from western and eastern CCA cohorts are needed.

## Data Availability Statement

The raw data supporting the conclusions of this article will be made available by the authors, without undue reservation.

## Ethics Statement

The studies involving human participants were reviewed and approved by the Liver Cancer Institute, Zhongshan Hospital, Fudan University. The patients/participants provided their written informed consent to participate in this study.

## Author Contributions

XY had full access to all the data in the study and takes responsibility for the integrity of the data and the accuracy of the data analysis. Study concept and design: BQ, ML, and XY. Acquisition, analysis, or interpretation of data: BQ, ML, SL, and JL. Drafting of the manuscript: BQ, WL, and FZ. Critical revision of the manuscript for important intellectual content: RC, ZR, and XY. Statistical analysis: BQ, ML, SL, and JL. Obtained funding: XY. Administrative, technical, or material support: RC, ZR, and XY. Study supervision: RC, ZR, and XY. All authors contributed to the article and approved the submitted version.

## Funding

This study was supported by the National Natural Science Foundation of China (No. 81972889) and Exploratory Clinical Research Projects of National Clinical Research Center for Interventional Medicine (2021-001).

## Conflict of Interest

The authors declare that the research was conducted in the absence of any commercial or financial relationships that could be construed as a potential conflict of interest.

## Publisher’s Note

All claims expressed in this article are solely those of the authors and do not necessarily represent those of their affiliated organizations, or those of the publisher, the editors and the reviewers. Any product that may be evaluated in this article, or claim that may be made by its manufacturer, is not guaranteed or endorsed by the publisher.

## Abbreviations

CCA, cholangiocarcinoma; iCCA, intrahepatic cholangiocarcinoma; pCCA, perihilar cholangiocarcinoma; dCCA, distal cholangiocarcinoma; PHx, partial hepatectomy; PD, pancreatoduodenectomy; OS, overall survival; DSS, disease-specific survival; ANN, artificial neural network; TB, total bilirubin; AKP, alkaline phosphatase; ALT, alanine aminotransferase; AST, alkaline phosphatase; γ-GT, γ-glutamyl transpeptidase; CA19-9, carbohydrate antigen 19-9; BDT, bile duct thrombi; CT, computerized tomography; MRI, magnetic resonance imaging; IQR, interquartile range; AJCC, American Joint Committee on Cancer; DCA, decision curve analysis; ROC, receiver operating characteristic; RF, random forest; AUC, area under the curve; CI, confidence interval.
